# A New Species of the Genus *Microhyla* (Amphibia: Anura: Microhylidae) from the Dabie Mountains, China

**DOI:** 10.3390/ani12212894

**Published:** 2022-10-22

**Authors:** Caiwen Zhang, Cheng Chen, Meihua Zhang, Zhiyue Wang, Haohao Ma, Ruolei Sun, Jianping Jiang, Baowei Zhang

**Affiliations:** 1 School of Life Sciences, Anhui University, Hefei 230601, China; 2 Chengdu Institute of Biology, Chinese Academy of Sciences, Chengdu 610041, China

**Keywords:** *Microhyla*, taxonomy, phylogenetic analyses

## Abstract

**Simple Summary:**

In China, new species of Microhylidae continue to be discovered at a rapid rate, often as a result of re-examining geographically widespread species populations using new molecular and bioacoustic tools. Here, we show that members of the genus *Microhyla* from the Dabie mountains in East China can be distinguished from morphologically similar species (*M. beilunensis*, *M. fanjinshanensis* and *M. mixtura*) with the analysis of phylogeny, species delimitation, bioacoustics and morphology. Based on the above multiple lines of evidence, we describe the population of the Dabie Mountains as a new species, *Microhyla dabieshanensis* sp. nov. This study enriches the diversity of Microhylidae, and clarifies the species of the genus *Microhyla* in the Dabie Mountains. *Microhyla dabieshanensis* sp. nov. is distributed in the Dabie Mountains above 500 m above sea level, and in some areas has sympatric distribution with *M. fissipes* and *M. heymonsi*.

**Abstract:**

Species belonging to the genus *Microhyla* are small-sized frogs that are widely distributed in southern, eastern, and south-eastern Asia. In China, the genus harbors many cryptic species, on two of which—*M. beilunensis* and *M. fanjinshanensis*—studies were recently published. In this study, we collected specimens from the Dabie Mountain range, which is at the junction of Anhui, Henan and Hubei Provinces, East China; these specimens belonged to a species previously identified as *M. mixtura*. Based on phylogenetic analyses, species delimitation analyses, morphological comparisons and advertisement calls comparisons, we found they were significantly different from other known congeners, and thus we describe them as a new species. This study enriches the diversity of Microhylidae, and clarifies the species of the genus *Microhyla* in the Dabie Mountains.

## 1. Introduction

The genus *Microhyla* Tschudi, 1838 (Amphibia: Anura: Microhylidae), currently contains 48 species [1], and many species are widely distributed across southern, eastern, and south-eastern Asia [2,3]. Over the last decade, there have been 25 newly described species [2,3,4,5,6,7,8,9,10]. Recent molecular systematics studies have shown that the taxon conceal numerous highly divergent lineages, which suggests that many hidden species have not been discovered [2,3,4,5,6,7,8,9,10].

In China, nine species (*M. beilunensis*, *M. berdmorei*, *M. butler*, *M. fanjingshanensis*, *Microhyla fissipes*, *M. heymonsi*, *M. mixtura*, *M. mukhlesuri* and *M. pulchra*) of *Microhyla* have been recorded so far, and are widely distributed in the south and southeast of China [11]. *M. beilunensis*, *M. fanjingshanensis* and *M. mixtura* are endemic to China [1]; *M*. *mixtura*, especially, has a wide distribution in China (Anhui, Henan, Guizhou, Hubei, Chongqing, Shaanxi, Sichuan and Zhejiang Provinces) [12,13]. Previous studies have found that *M. mixtura* in different geographical groups have differences in morphology such as skin texture, patterns and spots, supratympanic fold and canthus rostralis [12,13,14].

From 2019 to 2021, we collected nineteen *Microhyla* specimens from the Dabie Mountains of Anhui Province, China, which superficially resembled *M. mixtura*. Based on phylogenetic analyses using DNA sequences of the mitochondria (12S rRNA, 16S rRNA and COI genes), the specimens more likely represent a cryptic species in this study. In addition, the subsequent morphological comparisons and bioacoustics studies all consistently suggested that the Dabie Mountains specimens were distinctly different from known *Microhyla* species, and we therefore describe these specimens as a new species here.

## 2. Materials and Methods

### 2.1. Sampling

**Figure 1:**
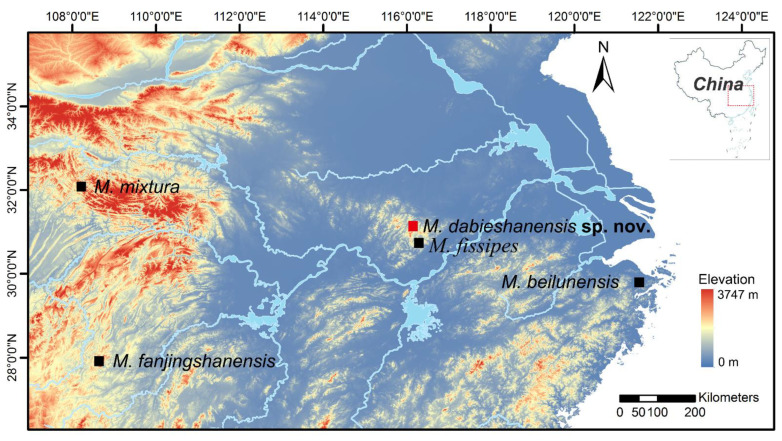
Data location in this study (China). (1) *Microhyla dabieshanensis* sp. nov., Dabie Mountain, Anhui Province; (2) *M. fissipe*, same location as new species; (3) *M. fanjingshanensis*, Fanjing Mountain, Guizhou Province; (4) *M. mixtura*, Hua’e Mountain, Sichuan Province; (5) *M. beilunensis*, Beilun District, Zhejiang Province.

Between May 2019 and September 2021, we collected 19 adult specimens, comprising 5 females and 14 males, from the Dabie Mountains of Anhui Province, China (Figure 1). In addition, one adult specimen of *M. fissipes* from Dabie Mountains of Anhui Province was collected for comparison. The specimens were euthanized, photographed and fixed in 4% formaldehyde for two days, and then finally washed and preserved in 70% ethyl alcohol. Voucher specimens for this work were deposited at the Anhui University Biology Museum (AHUBM). Voucher specimens were deposited at the Museum of Anhui University.

### 2.2. Molecular Phylogenetic Analyses

Tissue samples from six individuals of the new taxon were used for the phylogenetic analyses. Total genomic DNA was extracted from muscle or liver tissue based on a standard phenol/chloroform extraction method [15]. For molecular analysis, the mitochondrial genes (12S rRNA, 16S rRNA and COI) of six samples were amplified and sequenced (see Table S1). The PCR procedures and primers we used were from previous studies [16,17,18]. Experimental primer pairs were synthesized and provided by General Biosystems (Anhui) Co., Ltd. (Anhui, China) The conditions for PCR amplification are as follows: initial denaturation step, 95 °C, 4 min; the second step is 35 cycles at 94 °C, 35 S, annealing at 46–52 points; then, expand at 72 °C for 1 min; finally, extend the steps for 10 min of 72 °C. PCR amplification products were sent to General Biosystems (Anhui) Co., Ltd. The final sequence was submitted to GenBank (GenBank Accession numbers shown in Table S1).

SeqMan software (version 7.1.0, Madison, WI, USA) was used to splice the sequences obtained by sequencing and check whether there were any errors, which were manually corrected. MEGA software (version 7.0, Mega Limited, Auckland, New Zealand) [19] was used to compare the sequenced and spliced sequences with the downloaded apochromat sequences and adjust the sequences appropriately.

### 2.3. Phylogenetic Analyses

Phylogenetic analyses, involving 12S rRNA-16S rRNA-COI sequences of 49 *Microhyla* species and 8 *Nanophla* species, were downloaded from GenBank and used for phylogenetic reconstruction. Phylogenetic trees were constructed using maximum likelihood (ML) and Bayesian inference (BI) analyses.

According to previous studies, we chose *Dyscophus antongilii* and *Kaloula verrucosa* as the outgroups [20]. Phylogenetic analysis was performed using maximum likelihood (ML) and Bayesian inference (BI) methods, which were implemented in PhyML (version 3.0, France Génomique) [21] and MrBayes (version 3.2.2, Stockholm, Sweden) [22], respectively. PAUP (version 4.9, Gainesville, FL, USA) [23] software was used to construct the Bayesian system analysis under the MRModeltest 2.0 [23] program, and determine the best model parameters (minimum-lnL) with the nucleotide alternative model and the replacement rate heterogeneity test based on the Akaike information criterion (AIC) parameter standard. The best model parameters were selected to modify the Bayes program running file format, and MrBayes3.2.2 software was used to construct a Bayes phylogenetic tree based on the Markov chain Monte Carlo (MCMC) algorithm [24]. The results showed that the GTR+I+G model was the best partition. In the BI analysis, four Markov chains were used for two runs over three million generations, sampling every 300 generations. The first 25% of generations were burned in, and the remaining trees were used to generate the 50% strictly consistent tree. The posterior probability (PP) of each node in the phylogenetic tree was also calculated. All trees had to have an error of less than 0.01. For ML trees, branch support was extracted from 10,000 nonparametric bootleg replicates. By default, tree nodes in the ML tree with bootstrap values of 75% or greater were considered sufficiently resolved [25,26].

Genetic distances between and within species were calculated using uncorrected *p*-distances with 16S rRNA implemented in MEGA v7.0.

### 2.4. Species Delimitation Analysis

Multilocus coalescent delimitation was run with the 12S rRNA, 16S rRNA and COI; genes. We conducted coalescent-based species delimitation analyses of the mitochondrial genes using BPP v3.4 [27]. We assigned the 21 individuals to 5 species, including the new taxon, *M. beilunensis*, *M. fanjingshanensis*, *M. okinavensis* and *M. mixtura*, based on the split results and Bayesian tree topology. With each algorithm (0 and 1), we analyzed the data in BPP under three different sets of gamma priors for population size (θ) and divergence time at the root of the species tree (τ0): (1) small ancestral population sizes and shallow interspecific divergences: θ ~ Γ (2, 1000), τ0 ~ Γ (2, 1000); (2) large ancestral population sizes and shallow interspecific divergences: θ ~ Γ (1, 10), τ0 ~ Γ (2, 1000); and (3) large ancestral population sizes and deep interspecific divergences: θ ~ Γ (1, 10), τ0 ~ Γ (1, 10). We performed species delimitation analyses using a fixed guide tree (A10) derived from the concatenated sequences, joint species delimitation and species tree estimation (A11) [27]. Finally, we ran 24 independent analyses using combinations of models A10 and A11, algorithms 1 and 2, and different priors to avoid mixing problems (Table 1).

### 2.5. Morphological Analyses

The terminology and methods followed Fei et al. (2009) [12]. Morphological measurements were taken with a digital caliper to the nearest 0.01 mm. A total of 16 morphological indicators were measured, and the abbreviations of morphological characteristics used in the text are as follows:
SVLsnout–vent length; HDLhead length;HDWmaximum head width; SNLsnout length; EDeye diameter;UEWupper eyelid widthIODinterorbital distance; INDinternasal distance;LALlength of lower arm and hand;LWlower arm width;HALhand length;HLLhindlimb length;TWtibia width;TFLlength of foot and tarsus and;FLfoot length.

We also compared the morphological characteristics of the collection species with all other *Microhyla* genera. Morphological data for comparisons were obtained from the relevant literatures [2,3,4,5,6,7,8,9,10,11,12,13,28,29,30,31,32,33,34,35,36,37,38,39,40,41,42,43,44,45,46,47,48,49,50,51,52,53,54,55,56,57,58,59,60,61,62,63,64,65,66].

The collection species, *M. beilunensis* and *M. fanjingshanensis*, were previously classified as *M. mixtura* (Wu et al., 1986; Fei et al., 2009, 2012). When comparing the population morphology characteristics of our collection species with other known species, it is important to compare these three species that once belonged to *M. mixtura* (*M. mixtura*, *M. beilunensis* and *M. fanjingshanensis*). Therefore, we focused on conducted morphological comparisons to explore the morphological differences between these four species. A total of 19 specimens of our collection taxon, comprising 14 males and 5 females from the Dabie Mountains, were measured. At the same time, data on 8 males of *M. mixtura* [4], 37 males and 4 females of *M. beilunensis* [4], and 14 males and 2 females of *M. fanjingshanensis* [5] were obtained from the available literature (voucher information: see Table S2).

To reduce the influence of allometric growth in afterward morphometric analyses, we calculated the size-correction value of each morphological trait as the ratio to SVL. Wilcoxon rank sum tests were conducted to test the significance of the differences of the morphometric characteristics between the different sexes, as well as between different species. The significance level was set at 0.05. Furthermore, we then employed principal component analysis (PCA) to explore the morphological differences between the populations of the Dabie Mountains—*M. beilunensis*, *M. fanjingshanensis* and *M. mixtura*—and checked whether the different species were consistently separated in morphological characteristics. All above analyses were carried out in R 4.0.2. All morphometric analyses were conducted separately for the male and female groups. Because we did not collect females of *M. mixtura*, only the collection taxon and *M. beilunensis* and *M. fanjingshanensis* females were included.

### 2.6. Bioacoustics Analyses

Ten calls from two individuals of the new taxon were recorded in the Dabie Mountains of Anhui, China (31.28° N and 115.72° E; elevation 871 m a. s. l.). For comparison, we collected the advertisement calls of *M. beilunensis* recorded in Suichang, Zhejiang (28.37 N, 118.90 E; elevation 1002 m a. s. l), the advertisement calls of *M. fanjinshanensis* recorded in the Fanjing Mountains (27.9153° N, 108.61026° E; elevation 1139 m a. s. l.) and the advertisement calls of *M. mixtura* recorded in the Hua’e Mountain (32.05 N, 108.37; elevation 1132 m a. s. l.) (Figure 1). Recording the subjects’ calls was conducted using a Sennheiser ME66 microphone (Sennheiser, Wedemark, Germany). The microphone must be placed 0.2 m from the subject for the best recording quality and mounted on a long bamboo rod to keep it at the right height. The microphone was connected to a laptop (Thinkpad X201; Lenovo, Beijing, China) with a sampling rate of 44.1 KHz and a 16-bit resolution. Adobe Audition 3.0 software (San Jose, CA, USA) was used to gather relevant data, such as amplitude-modulated waveforms (oscillograms) and audio spectrograms of male advertisement calls. The methods for data analysis were similar to those described previously [67]. The ambient temperature of the type locality was taken by a digital hygrothermograph.

## 3. Results

### 3.1. Phylogeny Analysis and Species Delimitation

**Figure 2:**
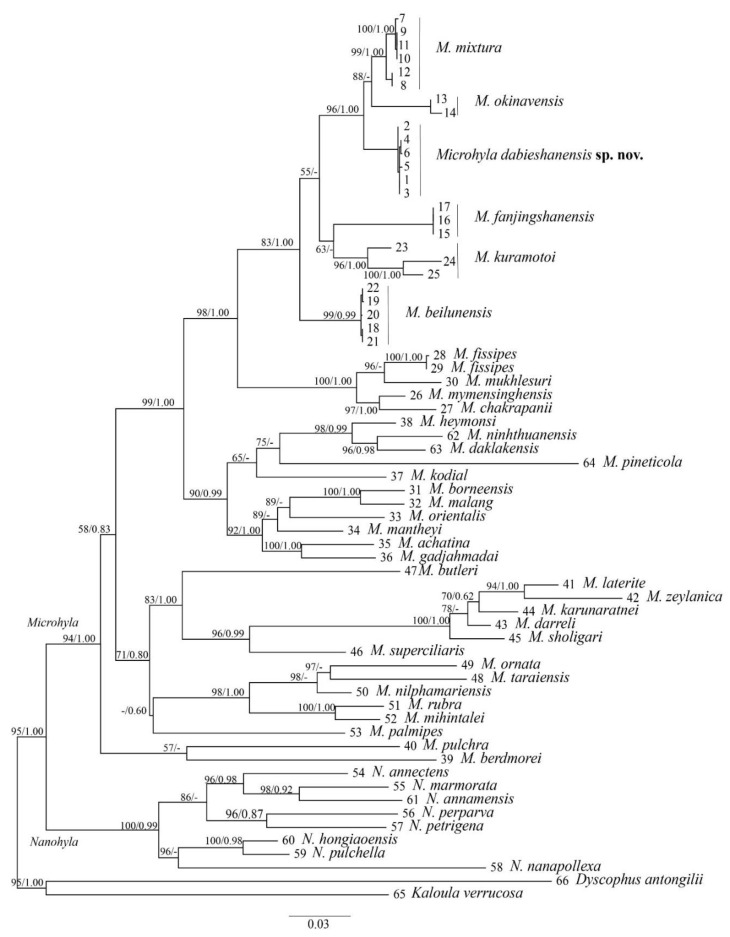
Maximum likelihood (ML) tree of the genus *Microhyla* reconstructed based on DNA sequences of segments of the 12S rRNA, 16S rRNA and COI genes. ML bootstrap support/Bayesian posterior probability are denoted beside each node, and the symbol “-” indicates value below 50%. See information on samples 1–66 in Table S1.

The aligned dataset contained 39 individuals of the *Microhyla* species, 8 individuals of the *Nanohyla* species and two individuals of the outgroup species (Table 1). The aligned matrix of the mitochondrial 12S + 16S + COI genes contained 1412 bp, of which 933 sites were conserved, 473 sites exhibited variation, and 379 were found to be potentially parsimony-informative. Maximum likelihood (ML) and Bayesian inference (BI) analyses obtained similar topologies (Figure 2) that differed only at some poorly supported basal nodes. This ML tree could be divided into two major branches based on topology display, corresponding to *Microhyla* and *Nanohyla*, respectively. In the phylogenetic tree, all six new samples were aggregated into a monophyletic group, which was sister to *M. mixtura* and *M. okinavensis* with high nodal support values (1.00/99). Overall, the phylogenetic relationship of *Microhyla* species obtained in this study is consistent with the results of previous related studies [2,4,5,7].

The genetic distances (uncorrected *p*-distance based on the 16S rRNA genes) between the new samples and their congeners ranged from 2.0% (vs. *M. okinavensis*) to 11.4% (vs. *M. zeylanica*) (Table S3). The genetic distances between the new samples and their close relatives (*M. beilunensis*, *M. fanjinshanensis*, *M. kuramotoi*, *M. mixtura* and *M. okinavensis*) ranged from 2.0% to 3.3%, and were higher than or equal to the values of various pairs of sister species.

Furthermore, we used BPP to test a five-species scenario in the subclade containing the new samples and four close relatives (*M. beilunensis*, *M. fanjinshanensis*, *M. mixtura* and *M. okinavensis*), and achieved results with high posterior probability-supported validity for all species (PP ≥ 0.98, Table 1), indicating that the new samples are distinct from all recognized species, thus supporting their recognition as a new taxon at the species level.

### 3.2. Morphology

**Figure 3:**
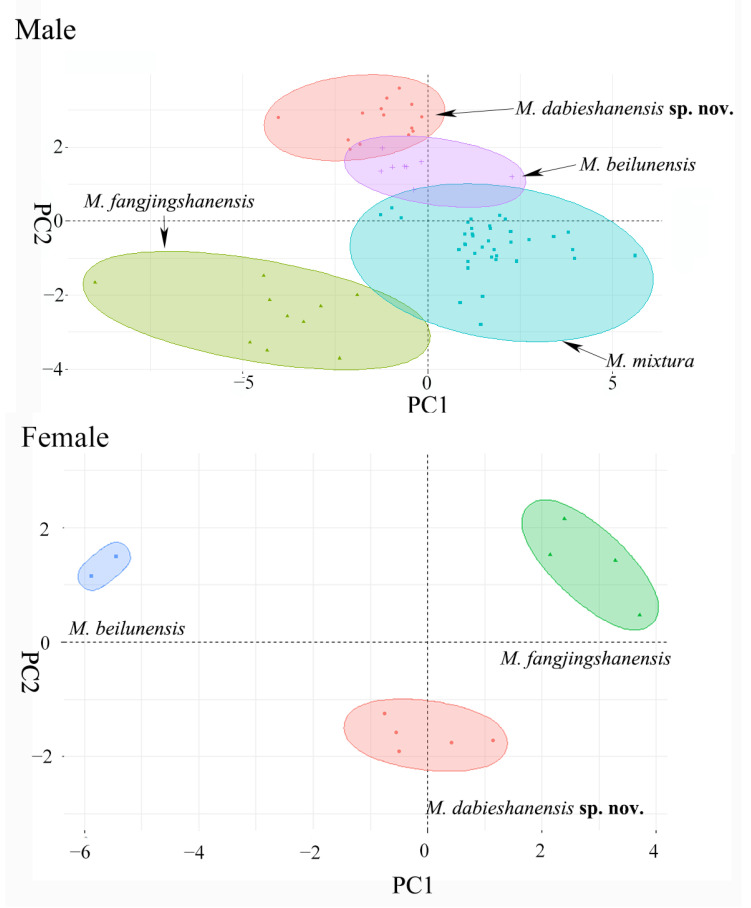
Plots of the first principal component (PC1) versus the second (PC2) for *Microhyla dabieshanensis* sp. nov., *M. beilunensis*, *M. fanjingshanensis* and *M. mixtura* from the principal component analysis. Above, male; under, female.

In morphological comparison, the results of the Wilcoxon rank sum test indicated that the new taxon male group was significantly different from those of *M. beilunensis*, *M. fanjingshanensis* and *M. mixtura* in many morphometric characteristics (all *p*-values < 0.05; Table S4). Unfortunately, the new taxon’s female group was not significantly different from those of *M. beilunensis* and *M. fanjingshanensis* according to the Wilcoxon rank sum test, in many morphometric characteristics (Table S4), which may be due to an insufficient number of samples. PCA extracted principal component factors with Eigenvalues greater than four in males and two in females. The first four principal components explained 81.72% of the total variation in the males, and the first two principal components explained 86.08% of the total variation in the females (Table 2). These results were mainly affected by morphological characters, including SVL, HDL, HDW, ED, LW, TL, TW and TFL (the corrected relevant values shown in Table S4). Both the male and female groups of the four aforementioned species can be clearly separated on the two-dimensional graphs of PC1 and PC2 (Figure 3).

### 3.3. Bioacoustics

**Figure 4:**
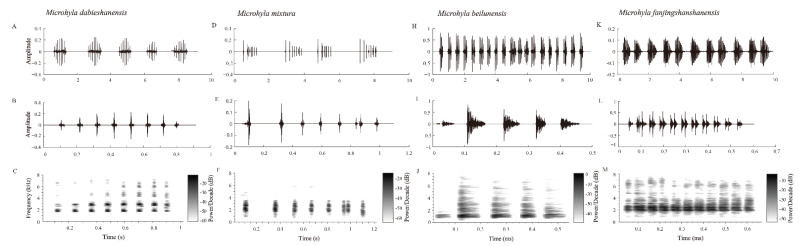
Sample recordings of the advertisement call of the four *Microhyla* species. (**A**, **D**, **H** and **K**) Oscillogram of a 10 s long call bout of the *M. dabieshanensis* sp. nov., *M. mixtura*, *M. beilunensis* and *M. fanjingshanensis*, respectively. (**B**, **E**, **I** and **L**) detailed view of one advertisement call of the oscillogram and (**C**, **F**, **J** and **M**) spectrogram of the same call of the four *Microhyla* species.

Ten advertisement calls of the new species were recorded in the Qingfengling Village, Huoshan County, Luan City, Anhui Province (Figure 1), on 22 April 2021 between 20:00 and 22:00. The advertisement description is based on recordings of AHU2021QFL01 (Figure 4) from a field near the streamlet at an ambient air temperature of 13 °C and air humidity of 90.8%. Each call had 6–9 notes (mean 7.5 ± 0.849, n = 10). The call duration was 0.598–0.863 s (mean 0.744 ± 0.083, n = 10), with intervals ranging from 1.231 to 2.253 s (mean 1.656 ± 0.347, n = 10). Amplitude modulation within each note was apparent, beginning with moderately high energy pulses, increasing to the maximum and then decreasing towards the end. The average dominant frequency was 2764 ± 102.96 (2670–2971 Hz, n = 10). At the same time, we also collected the acoustic data of the new specimen’s close relative species (*M. beilunensis*, *M. fanjingshanensis* and *M. mixtura*) distributed in China.

There were three differences in the sonograms and call waveforms among the new species and its close relative species, *M. beilunensis*, *M. fanjingshanensis* and *M. mixtura* (Figure 4; Table 3). The number of notes called by the new species (mean 7.5 ± 0.8) was lower than those of *M. mixtura* (mean 8.5 ± 0.8) and *M. fanjingshanensis* (mean 10.4 ± 0.8), but was higher than that of *M. beilunensis* (mean 6.0 ± 1.8). The calling rates of the new species (25.0/min) were very different from *M. beilunensis* (112.1/min) and *M. fanjingshanensis* (16.4/min), and the call of the new species had higher dominant frequencies (2670–2971 Hz) than other species.

These three main differences in bioacoustics can be distinguished from the three close relative species, *M. beilunensis*, *M. fanjingshanensis* and *M. mixtura*, and support the new specimen as a new species.

### 3.4. Taxonomic Account

The results of the molecular phylogenetic analyses, morphological comparison and bioacoustics all indicated that the new taxon of the genus *Microhyla* from the Dabie Mountains is significantly different from other known species of the same genus. Therefore, we describe it here as a new species.

*Microhyla dabieshanensis* sp. nov. Zhang, Chen et Zhang.

http://zoobank.org/urn:LSID: zoobank.org:urn:lsid:zoobank.org:act:E5764271-28BC-4741-88B0-7072961307F0 (accessed on 1 October 2022).

**Figure 5:**
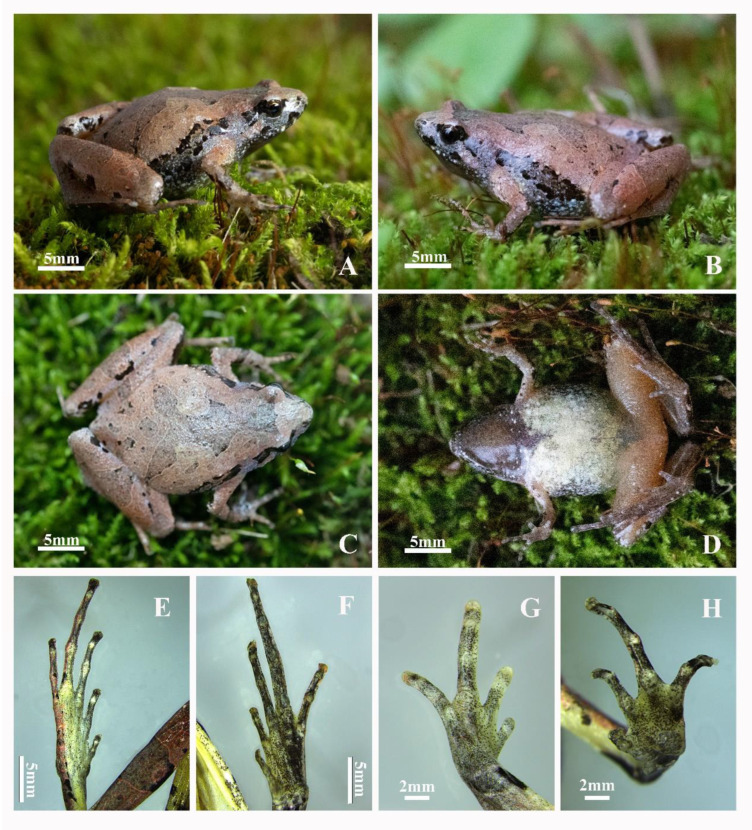
Photos of the holotype AHU2021QFL01 of Microhyla dabieshanensis ***sp. nov****. (***A**) right *lateral* view; (**B**) left *lateral* view; (**C**) dorsal view; (**D**) ventral view; (**E**) dorsal view of foot; (**F**) ventral view of foot; (**G**) dorsal view of hand; (**H**) ventral view of hand.

Holotype: AHU2021QFL01 (Figure 5), adult male, collected by C. W. Zhang and H. H. Ma in Qingfengling (31°10′49.22″ N, 116°13′56.81″ E; elevation 635 m a. s. l.), Foziling Provincial Nature Reserve, Huoshan County, Luan City, Anhui Province, China on 11 May 2021.

**Figure 6:**
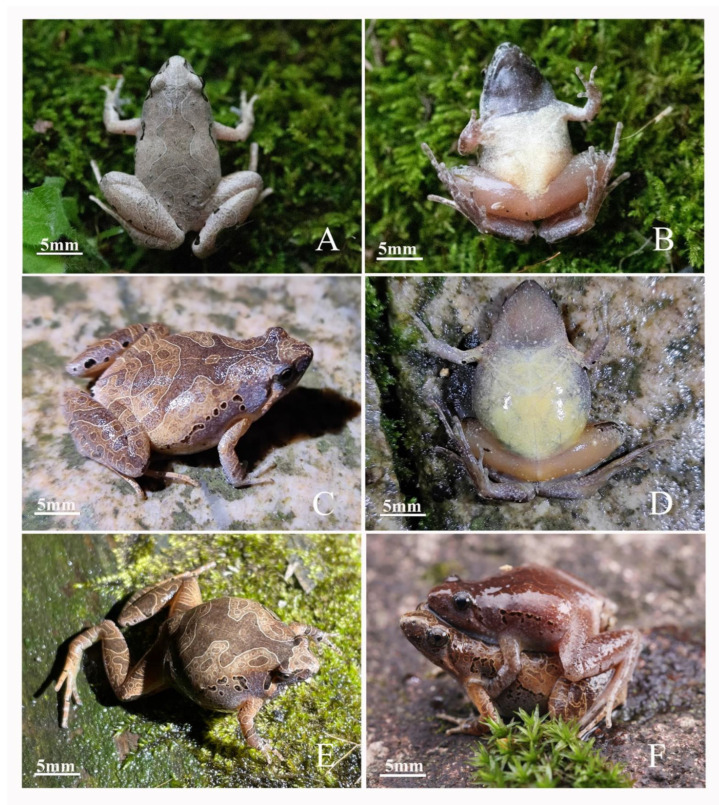
Color variation. (**A**–**D**) male; (**E**) female; (**F**) couple mating; (**A**) dorsal view; (**B**) ventral view; (**C**) dorsal view; (**D**) ventral view; (**E**) right dorsal lateral view.

Paratypes: AHU2021QP01-07, five males and two females were collected by C.W. Zhang, C. Chen and H.H. Ma on 2 May 2021 from the Tianma National Nature Reserve, Jinzhai County, Luan City, Anhui Province (Figure 6).

Etymology: The specific *Microhyla dabieshanensis* sp. nov. refers to the distribution of the new species in the Dabie Mountains in China. We recommend the English name would be the “Dabie Mountains Pygmy frog” and Chinese name as “大别山姬蛙 (Dà Bié Shān Ji Wā).”

#### 3.4.1. Diagnosis

*Microhyla dabieshanensis* sp. nov. is distinguished from its congeners by some morphological characteristics: (1) medium body size (SVL 19.1–22.5 mm, n = 14 males; 24.9–26.7 mm, n = 5 females); (2) relatively smooth dorsum skin; (3) absent disks and dorsal median longitudinal grooves on finger tips; (4) rudimentary webbing in toe; (5) toe tips have present disks and dorsal median longitudinal grooves, except for toe I; (6) two palmar tubercles, the outside is slightly larger than the inside; (7) when the leg is stretched forward, tibiotarsal articulation could reach the middle of the eye to the nostril; (8) in the upper middle of the chest, many individuals have a vaguely visible V-shaped white stripe.

#### 3.4.2. Holotype Description

Adult males have a medium body size (SVL 20.9 mm), with a head wider than its length (HDL/HDW 83.6%), a snout short that is rounded, projecting beyond the lower jaw, and distinct rounded nostrils that are obviously closer to the tip of the snout than the eye. The interorbital distance (2.4 mm) is greater than the internasal distance (1.7 mm), and the upper eyelid width is 1.6 mm with an eye diameter of 2.2 mm. The eyes are small and slightly protuberant; their diameter makes up 43.1% of head length, and the pupils are round. This species also has hidden tympanum, with inconspicuous canthus rostralis and supratympanic fold. Their tongue is posteriorly oval and not notched behind, and they do not have maxillary or vomerine teeth.

Their forelimbs are relatively short and thin—about four times shorter than the snout–vent length (25.8% of SVL), and their fingers are short and slender, distally rounded, unwebbed, and have disks but absent dorsal longitudinal grooves. The relative finger lengths are: I < II < IV < III. There are distinct subarticular tubercles that are roughly circular. The supernumerary tubercles below the base of the finger are absent, although there are two palmar tubercles, with an elliptical inner palmar tubercle and round outer palmar tubercle slightly larger than the inner palmar tubercle (Figure 5H). The nuptial pads are absent.

The hindlimbs are slender and comparatively long (HLL/SVL 189.5%), the tibia length is longer than half of the snout–vent length (TL/SVL 54.8%) and one-third of the hindlimb length (TL/HLL 29.0%). The heels overlap when the hindlimbs are at a right angle to the body, and the tibiotarsal articulation of the adpressed limb reaches the level between the eye and nostril when the leg is stretched forward. The foot length is equal to that of the tibia (FL/TL 1), and the relative toe lengths are: I < II < V < III < IV. The toes are distally rounded, equipped with disks and longitudinal grooves except for toe I, and the toes with have rudimentary webbing with absent supernumerary tubercles.

The dorsal skin surfaces of the head and body are slightly smooth with sparse tiny tubercles in the cloacal region. There are also thigh granules, although the throat, chest, and ventral part of the thigh and tibia are smooth.

#### 3.4.3. Holotype Coloration

The dorsal body is reddish brown or grayish, with obvious dark brown markings and a light brown outline. A thick, dark brown inverted triangle marking sits between the eyes and on the dorsum, along with three pairs of symmetrical figures or stripes where the anterior pair of figures are shorter and extend laterally to the middle of the upper eyelid; three pairs connect to each other, with the middle pair being relatively long and extending laterally to the shorter. The posterior pair of stripes are the longest, running obliquely from above the shoulder to the hip; the dorsum of the body’s posterior part, including the legs, is brownish, and scattered with dense, rounded dark brown irregular spots, as well as bar-shaped patterns and streaks with light margins; the supratympanic fold is dark brown; there are more continuous dark stripes on the side; the limbs are indefinitely barred in dark brown; the throat is dark purple, and the chest and belly are white-mottled with black.

#### 3.4.4. Intraspecific Morphological Variations

The 14 adult male and 5 adult female specimens had nearly similar morphological features. The basic statistics of morphological measurement are shown in Table S2. However, the dorsum coloration and the color and shape of the stripes were considerably different between individual specimens (Figure 6). In some specimens, the stripes on the dorsum were shallow or narrow, and asymmetrical or discontinuous in the posterior pair of stripes. Light-colored spots were unclear on the venter in some individuals. Many adult male individuals have a white-mottled brown venter and a vaguely visible V-shaped white stripe on the upper midsection. Some individuals have a round light spot in the middle of the stripe on the dorsum.

#### 3.4.5. Sexual Dimorphism

Adult female snout–vent length is significantly larger than that of adult males. Adult males have slit-like openings to a median subgular vocal sac, and absent nupital pads and spines.

#### 3.4.6. Distribution and Habit

To date, *Microhyla dabieshanensis* sp. nov. is only known from its type locality in the Dabie Mountains, China (Figure 1), and this species’ distribution is in the water ditches or rice fields or adjacent grass thickets at elevations of 535–1300 m a. s. l., sympatric with *M. fissipes* and *M. heymonsi*.

#### 3.4.7. Morphological Comparisons

*Microhyla dabieshanensis* sp. nov. is morphologically most like *M. beilunensis*, *M. fanjingshanensis*, *M. kuramotoi*, *M. mixtura* and *M. okinavensis*. However, *Microhyla dabieshanensis* sp. nov. could be distinguished from these species via smoother skin with few tubercles and granules, and the canthus rostralis and supratympanic fold were not obvious.

*Microhyla dabieshanensis* sp. nov. differs from *M. beilunensis* in that it has a vaguely visible V-shaped stripe in the upper middle of the chest (vs. without stripe), higher ratios of HDL, SL, LAL, LW, HAL, HLL, TFL and FL to SVL (*p*-values < 0.05; Table S4).

*Microhyla dabieshanensis* sp. nov. differs from *M. fanjingshanensis* as it has: (1) a longer SVL (24.9–26.7 mm) in females (vs. SVL 22.5–23 mm); (2) a vaguely visible V-shaped stripe (vs. stripe obvious of each individual); and (3) significantly higher ratios of IOD and HL to SVL (*p*-values < 0.05; Table S4).

*Microhyla dabieshanensis* sp. nov. significantly differs from *M. kuramotoi* as follows: (1) the SVL is smaller, at around 19.1–22.5 mm in males and 24.9–26.7mm in females (vs. SVL 23.8–27.8 mm in males and 29.6 mm in females; (2) the vaguely visible V-shaped stripe (vs. without stripe); (3) toe I has no disk and dorsal median longitudinal groove on the tip (vs. all toes have); (4) in advertisement calls, *Microhyla. dabieshanensis* sp. nov. composes each note of 6–9 short pulses lasting for 0.598–0.863 s with a dominant frequency at 2.7–3.0 kHz (vs. each note with 4–8 pulses, with a duration of 0.11–0.21 s, and an average dominant frequency of 2.3–2.5 kHz in *M. kuramotoi*).

*Microhyla dabieshanensis* sp. nov. is distinct from *M. mixtura*, as it has: (1) the vaguely visible V-shaped stripe (vs. without the stripe); (2) posterior pairs of stripes on the dorsum with the longest on the belly, which is white-mottled mixed brown (vs. white); (3) higher ratios of HDL, SL, UEW, LW and HLL to SVL, and lower ratios of ED and TL to SVL in males (all *p*-values < 0.05; Table S4).

*Microhyla dabieshanensis* sp. nov. differs from *M. okinavensis* in the following ways: (1) the SVL is 19.1–22.5 mm in males and 24.9–26.7 mm in females (vs. SVL 22.5–28.2 mm in male and 26.5–30.8 mm in females); (2) the vaguely visible V-shaped stripe (vs. without the stripe absent); (3) the belly is white-mottled with brown (vs. white); (4) toe I has no disk and a dorsal median longitudinal groove on the tip (vs. all toes have).

*Microhyla dabieshanensis* sp. nov. can be distinguished from all other species of *Microhyla*, except *M. butleri*, *M. chakrapani*, *M. daklakensi*, *M. fissipes*, *M. fodiens*, *M. fusca*, *M. gadjahmadai*, *M. heymonsi*, *M. neglecta*, *M. ornata* and *M. rubra*, by different body size (SVL 19.1–22.5 mm in male and 24.9–26.7 in female) (Table 4). *Microhyla dabieshanensis* sp. nov. differs from *M. butleri* in the following ways: (1) fingers without disks and longitudinal grooves; (2) two metacarpal tubercles on palm (vs. three metacarpal tubercles). *Microhyla dabieshanensis* sp. nov. differs from *M. chakrapanii* by dorsal median longitudinal grooves on the toe disks except for the toe I (vs. grooves absent). *Microhyla dabieshanensis* sp. nov. differs from *M.* *daklakensi* by having purple–black throat (vs. white with scattered dark grey dusting on the throat). *Microhyla dabieshanensis* sp. nov. differs from *M. fissipes* by having: (1) disks on the toe tips except for toe I (vs. fingers and toes have no disks, white to whitish or cream ventral coloration on throat and belly); (2) a purple–black throat (vs. white throat); (3) a white belly mottled with brown (vs. white to cream ventral coloration on belly). *Microhyla dabieshanensis* sp. nov. differs from *M. fodiens* by having three pairs of symmetrical figures or streaks on the dorsum that connect to each other (vs. beige–brown “teddy-bear-shaped” streaks on the dorsum). *Microhyla dabieshanensis* sp. nov. differs from *M. fusca* by having disks and longitudinal grooves on the toe tips except for toe I (vs. both fingers and toes having disks and longitudinal grooves). *Microhyla dabieshanensis* sp. nov. differs from *M. gadjahmadai* by having a purple–black throat (vs. white throat). *Microhyla dabieshanensis* sp. nov. differs from *M. heymonsi* and *M. neglecta* by having: (1) a V-shaped stripe on the upper midsection (vs. without a V-shaped stripe); (2) no disks and longitudinal grooves on the fingers (vs. disks and longitudinal grooves on fingers). *Microhyla dabieshanensis* sp. nov. differs from *M. ornata* by having: (1) dorsal median longitudinal grooves on the toe disks except for toe I (vs. dorsal median longitudinal grooves and disks absent on toes tip); (2) the V-shaped stripe (vs. without the V-shaped stripe); and (3) the chest and belly white-mottled with reddish brown (vs. white to cream ventral). *Microhyla dabieshanensis* sp. nov. differs from *M. rubra* by having: (1) disks only on the toe tips except for toe I (vs. fingers and toes have no disks); (2) dark brown throat (vs. white throat); and (3) a white belly mottled with brown (vs. white to cream ventral coloration on throat and belly).

## 4. Discussion

In this study, a new species, *Microhyla dabieshanensis* sp. nov., was described in the Dabie Mountains area, China, which had previously been identified as *M. mixtura* [14]. In fact, *M. dabieshanensis* sp. nov. should represent an independent lineage distinctly separate from *M. mixtura*, *M. okinavensis* and other congeners in the phylogeny tree (Figure 2). In addition, it owns distinctive morphological characteristics and advertisement calls (Figure 3 and Figure 4).

The discovery of *Microhyla dabieshanensis* sp. nov. has brought the total number of known species in the genus *Microhyla* to 49 and the number of species in China to 10 [1,11]. Therefore, based on our results and those of previous studies [4,5,8,12,13], we could further complete distribution conclusions on these six phylogenetically closely related species. Overall, *M*. *dabieshanensis* sp. nov., *M. beilunensis*, *M. fanjingshanensi*, *M. okinavensis*, *M. kuramotoi* and *M. mixtura* have isolated distributions in the Dabie Mountains of Anhui Province, the Coastal Mountains of Zhejiang Province of China, the Wuling Mountains range of China, the Yaeyama Island, Ryukyu islands (Miyako, Okinawa and Amami Islands) and the Qinling–Daba Mountain range of China, respectively.

According to the distribution information of the collection sites of the 19 specimens, it is speculated that *M. dabieshanensis* sp. nov. is distributed throughout the Dabie Mountains above 500 m above sea level. According to our current survey results, three species of *Microhyla*, *M. dabieshanensis* sp.nov., *M. fissipes* and *M. heymonsi* are sympatric in the Dabie Mountains area. Therefore, further research is needed to elucidate the true distribution range and ecological niche of the new species, which is crucial for studying the interspecific relationships and sympatric distributions of the three *Microhyla* species in this area.

## 5. Conclusions

We described a new species of the microhylid frogs, *Microhyla dabieshanensis* sp. nov., from the Dabie mountains in East China, using an integrative taxonomic approach. *Microhyla dabieshanensis* sp. nov. can be differentiated from other congener species, *M. beilunensis*, *M. fanjingshanensis*, *M. kuramotoi*, *M. mixtura* and *M. okinavensis*, on the basis of phylogenetic analysis, species delimitation analyses, morphological characteristics and bioacoustics information. At the same time, we summarize the distribution areas of the six phylogenetically closely related species (*Microhyla dabieshanensis* sp. nov., *M. beilunensis*, *M. fanjingshanensis*, *M. kuramotoi*, *M. mixtura* and *M. okinavensis*). In the Dabie Mountains, *Microhyla dabieshanensis* sp. nov., *M. fissipes* and *M. heymonsi* are sympatric in some areas. Further research is needed to elucidate the true distribution range and ecological niche of the new species, as well as the interspecific relationships of *Microhyla* species in this area.

## Figures and Tables

**Table 1:** Evaluating a 5-species scenario including all samples. The mitochondrial concatenated gene tree was used as the guild tree.

**A10 (Species Delimitation Using a User-Specified Guide Tree)**						
nDNA-algorithm0	ε = 1	ε = 2	ε = 5	ε = 1	ε = 2	ε = 5
	heredity = 0	heredity = 0	heredity = 0	locusrate = 0	locusrate = 0	locusrate = 0
posterior probability [number of species]	P [5] = 0.99	P [5] = 1	P [5] = 0.99	P [5] = 1	P [5] = 0.99	P [5] = 0.99
nDNA-algorithm1	(α m) = (1 0.5)	(α m) = (1.5 1)	(α m) = (2 2)	(α m) = (1 0.5)	(α m) = (1.5 1)	(α m) = (2 2)
	heredity = 0	heredity = 0	heredity = 0	locusrate = 0	locusrate = 0	locusrate = 0
posterior probability [number of species]	P [5] = 1	P [5] = 0.99	P [5] = 0.99	P [5] = 1	P [5] = 0.99	P [5] = 1
**A11 (Joint Species Delimitation and Species Tree Inference)**						
nDNA-algorithm0	ε = 1	ε = 2	ε = 5	ε = 1	ε = 2	ε = 5
	heredity = 0	heredity = 0	heredity = 0	locusrate = 0	locusrate = 0	locusrate = 0
posterior probability [number of species]	P [5] = 0.99	P [5] = 0.99	P [5] = 0.99	P [5] = 1	P [5] = 0.98	P [5] = 0.99
nDNA-algorithm1	(α m) = (1 0.5)	(α m) = (1.5 1)	(α m) = (2 2)	(α m) = (1 0.5)	(α m) = (1.5 1)	(α m) = (2 2)
	heredity = 0	heredity = 0	heredity = 0	locusrate = 0	locusrate = 0	locusrate = 0
posterior probability [number of species]	P [5] = 0.99	P [5] = 0.99	P [5] = 0.99	P [5] = 1	P [5] = 0.99	P [5] = 0.99

**Table 2:** Variable loadings for principal components with Eigenvalue greater than 1, from morphometric characters corrected by SVL (all measurements were given in mm).

Morphometric Characteristics	Male				Female	
	PC1	PC2	PC3	PC4	PC1	PC2
SVL	0.205	−0.035	0.093	0.641	−0.269	−0.098
HDL	−0.103	−0.462	0.153	0.057	0.203	−0.392
HDW	−0.155	−0.405	0.222	−0.017	0.292	−0.162
SL	−0.325	0.148	0.117	−0.046	0.264	0.255
IND	−0.191	−0.107	0.39	−0.296	0.274	−0.23
IOD	0.178	−0.278	−0.415	−0.281	0.006	0.447
UEW	−0.199	0.416	0.145	−0.068	0.243	−0.194
ED	0.229	−0.277	−0.176	−0.297	0.094	−0.491
LAL	−0.288	0.036	−0.356	−0.152	0.263	0.227
LW	−0.247	−0.058	0.355	−0.232	0.273	0.222
HAL	−0.283	−0.038	−0.291	−0.224	0.276	0.061
HLL	−0.325	0.216	−0.088	0.049	0.253	0.295
TL	−0.261	−0.252	−0.22	0.287	0.286	−0.084
TW	−0.172	−0.365	0.2	0.116	0.29	−0.095
TFL	−0.332	−0.036	−0.28	0.258	0.247	0.068
FL	−0.346	−0.101	−0.129	0.18	0.279	0.033
Eigenvalues	6.605	3.753	1.559	1.158	10.751	2.022
Percentage of total variance	41.28	23.458	9.745	7.235	67.196	1.889
Cumulative percentage	41.28	64.738	74.483	81.718	61.196	86.083

**Table 3:** Descriptive statistics (means ± SD and range) for acoustic characteristics of advertisement calls in the four *Microhyla* species.

	Species	*M.dabieshanensis* sp. nov	*M. mixtura*	*M. beilunensis*	*M. fanjingshanensis*
Call Parameters					
Notes per call		7.5 ± 0.8	8.5 ± 0.8	6.0 ± 1.8	10.4 ± 0.8
		6–9	7–10	4–10	9–11
Call duration (s)		0.744 ± 0.083	1.048 ± 0.192	0.261 ± 0.069	0.574 ± 0.022
		0.598–0.863	0.872–1.422	0.195–0.444	0.872–1.422
Call interval (s)		1.656 ± 0.347	1.402 ± 0.390	0.274 ± 0.028	3.087 ± 0.065
		1.231–2.253	0.788–1.869	0.238–0.320	3.005–3.198
Call rate (call/min)		25.0	24.5	112.1	16.4
Dominant frequency (Hz)		2764.0 ± 102.9	2137.7 ± 70.6	2049.7 ± 291.3	2391.9 ± 69.3
		2670–2971	2024–2239	1730–2399	2306–2501

**Table 4:** Ranges of adult SVL (snout–vent lengths) of members of the genus *Microhyla* (all measurements are given in mm).

	Species	SVL			Species	SVL	
		Male	Female			Male	Female
1	*M. dabieshanensis* sp. nov	19.1–22.5	24.9–26.7	25	*M. maculifera*	**12.0–13.3**	**11.8**
2	*M. achatina*	**16**	**23**	26	*M. malang*	18.7–22.2	**19.0–23.4**
3	*M. aurantiventris*	**25.2–27.0**	**30**	27	*M. mantheyi*	15.0–29.2	**14.8–24.1**
4	*M. beilunensis*	19.08–23.73	26.39–28.25	28	*M. mihintalei*	21.7–27.3	**24.4**
5	*M. berdmorei*	**23.8–28.9**	26.2–45.6	29	*M. minuta*	**14.7–15.9**	**15.7–17.2**
6	*M. borneensis*	**10.6–12.8**	**17.9–18.8**	30	*M. mixtura*	20.5–23.7	23.8–26.6
7	*M. butleri*	20.0–25.0	21.0–26.0	31	*M. mukhlesuri*	16.5–21.0	**17.3–18.4**
8	*M. chakrapanii*	22	?	32	*M. mymensinghensis*	**14.2–17.6**	**15.2–21.3**
9	*M. daklakensi*	17.7–20.1	22.9–26.8	33	*M. nilphamariensis*	14.8–20.0	**18.7–21.0**
10	*M. darevskii*	**27.0–32.6**	?	34	*M. ninhthuanensis*	**17.3–18.8**	**21.6–23.6**
11	*M. darreli*	**15.0–15.7**	?	35	*M. okinavensis*	**22.5–28.2**	26.5–30.8
12	*M. fanjingshanensis*	19.0–22.7	**22.5–23.0**	36	*M. orientalis*	**15.8–17.4**	**15.8–17.4**
13	*M. fissipes*	18.0–27.5	20.0–28.0	37	*M. ornata*	13.4–24.9	24.9–26.2
14	*M. fodiens*	12.6–20.8	?	38	*M. palmipes*	**16**	**21.8**
15	*M. fowleri*	**29.5–32.5**	**32.2–41.5**	39	*M. picta*	**25.2–30.1**	**27.2–33.4**
16	*M. fusca*	23.0 **		40	*M. pineticola*	**16.0–18.0**	22.9–26.8
17	*M. gadjahmadai*	18.20–21.32	20.37–25.51	41	*M. pulchra*	**23.0–32.0**	**28.0–36.5**
18	*M. heymonsi*	16.5–22.0	18.0–26.5	42	*M. pulverata*	17.5–19.5	**18.8–20.2**
19	M. irrawaddy	**12.3–17.1**	**16.7–20.9**	43	*M. rubra*	20.0–27.5	20.5–29.5
20	*M. karunaratnei*	**15.8–19.1**	**19.6–21.0**	44	*M. sholigari*	?	**11.0–15.0**
21	*M. kodial*	**16.9–17.4**	**18.0–20.4**	45	*M. superciliaris*	?	**12**
22	*M. kuramotoi*	**23.8–27.8**	**29.6**	46	*M. taraiensis*	19.9–20.9	**22.1–24.9**
23	*M. neglecta*	18.7–20.2	23.4–26.2	47	*M. zeylanica*	**14.4–18.3**	**15.8–20.0**
24	*M. laterite*	**15.3–16.6**	**18.4**				

**Note:** ** indicates gender unknown; ? indicates no relevant data; species whose SVL ranges do not overlap with the new species are marked in bold.

## Data Availability

The data presented in this study are available on request from the corresponding author.

## Supplementary Materials

The following supporting information can be downloaded at: https://www.mdpi.com/article/10.3390/ani12212894/s1, TableS1. Specimens of Microhyla and Nanohyla used in the morphological and molecular analyses. Table S2. Measurements of the adult specimens of *Microhyla dabieshanensis* sp. nov., *M. fanjingshanensis*, *M. mixtura* and *M. beilunensis*. Table S3. Uncorrected p-distance on 16S rRNA gene between *Microhyla* species included in the phylogenetic analyses (below the diagonal), and standard error estimates (above the diagonal). Table S4. Morphological comparison of *Microhyla dabieshanensis* sp. nov. (MD) with *M. mixtura*, *M. beilunensis* (MB) and *M. fanjingshanensis* (MF).
